# The Feet

**Published:** 1890-01

**Authors:** 


### THE FEET.

A skillful anatomist says that if we wore shoes day and night our feet would become permanently and hopelessly diseased, but the airing and change they get while we sleep keep them in tolerable condition.

"The human foot is merely a hand modified for a base of structure to support the body." It is longer and thicker and narrower than the hand. It solid parts are firmer than the corresponding parts of the hand; the movable parts less movable than those of the hand. The foot has two arches; one from front to rear composed of eight bones, and another from side to side composed of four. These arches, on account of the cartilages interposed between the segments that compose them, are flexible and give elasticity to the step and gracefulness to the gait. The largest bone in the long arch of the foot is the heel bone, and to this is attached the largest tendon in the body. In this tendon the three muscles which compose the calf of the leg, and which are of the greatest value to us in the act of walking, unite.

The more nearly the shoe approaches the form of the foot, the easier it will be to walk in. High heels are nothing but an injury, not to the foot alone, but to the whole body. They flex the three muscles in the calf of the leg that give erectness of the body, throw the weight of the body on to the ball of the foot, throw the knees forward, and put the whole mechanism out of poise. This is well understood by lovers of field sports and athletics, whose shoes have hardly any heels at all.

The earliest form of foot cover was the simple sandal, secured to the foot by thongs, and often by a button, coming between the first and second toes. The material used for shoes and sandals is various, chiefly the skins of animals. Wooden shoes are much worn in Europe, and are becoming common in this country. The Japanese wear sandals of straw, and South Americans, in some localities, sandals of plaited hemp. The early Greeks went barefoot, or wore simple sandals; the Romans work buskins, similar to the moccasins of the American Indians.

The skillful shoe maker or shoe-fitter should understand the anatomy of the foot as well as the art of making shoes, and he should be able to fit each shoe to the foot that is to wear it, but probably not one shoemaker in a million ever dissected a human foot with a view to learning how shoes should be made.

We never think of working with our hands when they are gloved. and all we ask of a glove is that it neatly fit the hand when at rest. But we never think of walking any distance in unshod feet, and what we want of shoes is not covering only, but aid in locomotion. Many a shoe is comfortable enough when one is sitting still, that becomes excruciating when one walks in it. Room is not given for the play of the various muscles of the foot, the arches are pressed out of shape, the circulation obstructed, and the exercise of walking, which should be delightful, becomes intolerable, and the gait which should be graceful and easy becomes limping and awkward.

Judging from the number of misshapen feet one sees when traveling on the horse cars and crossing the ferries, where the feet of wayfarers are exposed to view, there is a great deal of suffering that is not much talked about, and is probably considered incurable. But it might all, or nearly all, have been prevented but for ill-fitting shoes. And a great deal of this suffering might be escaped if misshapen feet were provided with shoes fitted to them and conformed to their present necessities.—*New York Advocate.*

